# The muscle mass, omega-3, diet, exercise and lifestyle (MODEL) study – a randomised controlled trial for women who have completed breast cancer treatment

**DOI:** 10.1186/1471-2407-14-264

**Published:** 2014-04-16

**Authors:** Cameron McDonald, Judy Bauer, Sandra Capra, Joseph Coll

**Affiliations:** 1 Centre for Dietetics Research, University of Queensland, Brisbane, Australia; 2 The Wesley Research Institute, Brisbane, Queensland, Australia

**Keywords:** Breast cancer, Omega-3 fatty acids, Body composition, Exercise, Lean body mass, Inflammation

## Abstract

**Background:**

Loss of lean body mass (LBM) is a common occurrence after treatment for breast cancer and is related to deleterious metabolic health outcomes [*Clin Oncol*, **22**(4)**:**281–288, 2010; *Appl Physiol Nutr Metab*, **34**(5)**:**950–956, 2009]. The aim of this research is to determine the effectiveness of long chain omega-3 fatty acids (LCn-3s) and exercise training alone, or in combination, in addressing LBM loss in breast cancer survivors.

**Methods/design:**

A total of 153 women who have completed treatment for breast cancer in the last 12 months, with a Body Mass Index (BMI) of 20 to 35 kg/m^2^, will be randomly assigned to one of 3 groups: 3g/d LCn-3s (N-3), a 12-week nutrition and exercise education program plus olive oil (P-LC) or the education program plus LCn-3s (EX+N-3). Participants randomised to the education groups will be blinded to treatment, and will receive either olive oil placebo (OO+N-3) or LCn-3 provision, while the N-3 group will be open label. The education program includes nine 60-75min sessions over 12 weeks that will involve breast cancer specific healthy eating advice, plus a supervised exercise session run as a resistance exercise circuit. They will also be advised to conduct the resistance training and aerobic training 5 to 7 days per week collectively. Outcome measures will be taken at baseline, 12-weeks and 24-weeks. The primary outcome is % change in LBM as measured by the air displacement plethysmograhy. Secondary outcomes include quality of life (FACT-B + 4) and inflammation (C-Reactive protein: CRP). Additional measures taken will be erythrocyte fatty acid analysis, fatigue, physical activity, menopausal symptoms, dietary intake, joint pain and function indices.

**Discussion:**

This research will provide the first insight into the efficacy of LCn-3s alone or in combination with exercise in breast cancer survivors with regards to LBM and quality of life. In addition, this study is designed to improve evidence-based dietetic practice, and how specific dietary prescription may link with appropriate exercise interventions.

**Trials registration:**

ACTRN12610001005044; and World Health Organisation Universal trial number: U1111-1116-8520.

## Background

Breast cancer is the predominant cancer diagnosed in women with 1.4 million new cases diagnosed worldwide in 2008 [1]. Modern treatment protocols have resulted in a 5-year survival rate of 85% to 90% in developed countries, with Australia’s reported at 89.4% in 2012 [2]. Following treatment for breast cancer, a majority of women experience significant body weight increases [3-5]. These changes unfortunately, are comprised of simultaneous lean body mass (LBM) loss and fat tissue gain [4-7]. Furthermore, LBM loss and fat mass gains have been shown to occur in the absence of total body weight change [8]. Data from breast cancer cohorts reveal that weight gain is most strongly associated with premenopausal status at diagnosis [4], those who experience menopause as a result of treatment [4,9], lower weight at diagnosis, lower levels of physical activity [10], and longer chemotherapy treatment [5]. Evidence from pharmacological trials indicate that initial use of [11], or switching to aromatase inhibitors from tamoxifen [12,13] increases LBM, possibly due to the alteration in sex steroid balance. The complete aetiology of general LBM loss in this population is unclear, however it appears to be associated with poorer metabolic outcomes, such as earlier onset of cardiovascular disease and metabolic syndrome related diseases [14,15].

Currently, no definitive recommendations can be made in regards to the ideal weight or weight change for women who have completed treatment for breast cancer. Epidemiological studies using weight or BMI have indicated that weight stability may confer benefits in terms of mortality [16-18]. Currently there have been no trials assessing mortality and the impact of body composition change (LBM and fat tissue), however results from shorter intervention trials indicate that intentional weight loss and increased activity can improve biochemical markers associated with cardiovascular disease [19-21] and conditions related to metabolic syndrome [19,21], which both account for significant morbidity and mortality in this population.

### Interventions to improve body composition in women diagnosed with breast cancer

A number of studies have assessed the impact of diet, exercise or combined therapies on body composition during or following treatment reporting mixed effects. From studies that have reported a high quality measure of body composition assessment (i.e. Dual-Energy X-ray Absorptiometry: DEXA; Air Displacement Plethysmography: ADP; Computed Tomography: CT-scan; and Magnetic Resonance Imaging: MRI) resistance training is most likely to cause an increase in LBM [22,23], aerobic training overall has had mixed effects on LBM [21,24-26], with most studies indicating no change. Two studies that prescribed a combination of resistance and aerobic training have shown an increase in LBM [27,28]. Considering that aerobic exercise has been associated with improved disease-free survival in breast cancer populations [29,30], a combination of resistance and aerobic two may promote LBM growth and survival benefits extending beyond the study timeline. Some data indicate LBM increases may be more likely in younger individuals, and separately, those taking aromatase inhibitors (AIs) [21,31]. Dietary energy restriction alone has resulted in significant body weight loss but also involves significant LBM loss [19], while combining nutrition and exercise prescription may help to preserve LBM during weight loss [32], and/or ameliorate fat tissue gain during weight stability [33].

### Exercise and nutrition trials during chemotherapy

Numerous uncontrolled and controlled trials have been conducted assessing change in body weight and/or body composition. Of these trials, 11 studies that have used a high quality measure of body composition have indicated mixed effects on lean body mass for different modalities. Exercise only interventions conducted during chemotherapy have indicated that resistance exercise training is probably required to realise an increase in LBM [22], while aerobic training alone has shown little to no impact on LBM change [26]. When Courneya et al (2007) confined their analysis to women with more advanced breast cancer (Stage IIb & IIIa) significant improvements were seen in the intervention group compared to control, these differences were not seen in those women with earlier stage disease (Stage 0-IIa) [31]. Comparatively, combined exercise and nutrition interventions during chemotherapy have typically shown no effect on LBM change [33-35]. Lack of LBM gains may be a result of the less intensive/structured exercise training components prescribed in combined trials.

### Exercise and nutrition trials after completion of chemotherapy

A larger literature exists describing effects of exercise and nutrition on LBM in women after they have completed treatment (up to 3 to 4 years post). Of the four [21,23-25] studies reporting a high quality measure of body composition after exercise alone, two aerobic exercise studies (one controlled, one uncontrolled) reported statistically non-significant trends in LBM change [24,25], while separate aerobic [21] and resistance training [23] trials indicated a significant increase in LBM compared to control groups (+0.8 kg vs -0.8 kg, p = 0.047 & +0.88 kg vs +0.02 kg, P = 0.008, respectively). After further analysis, Irwin et al [36] found that exercisers aged <56 years had greater LBM gains than women >56 years and non-exercisers, and those taking AIs and exercising had greater LBM increases than those not taking AIs.

One well-designed study investigated dietary energy restriction alone on body composition and examined the differing effects of a low energy and low fat intake or low energy and low carbohydrate intake [19]. Both groups lost a similar and significant amount of body weight (6.1 kg + 4.8 kg) over 12 months, unfortunately this body weight change occurred at the expense of fat tissue and LBM. Incidence of sarcopenia, as defined by an appendicular LBM of <5.67 kg/m^2^, increased from 10% at baseline to 18% at the end of the trial [19,37].

Of the two studies that have assessed the effect of exercise and nutrition combined on LBM, one study has shown that LBM may be preserved by exercise during dietary energy restriction [32], while the other indicated a reduction in fat accumulation with no change to LBM during dietary energy balance [32,33,38]. After a 2000-4000kJ energy restriction plus a combined aerobic and resistance training protocol, Mefferd et al (2007) noted stable LBM in both intervention and wait-list control groups, however compared to control, the intervention group had a significant reduction in total body weight (-0.5 kg vs. -5.7 kg, p < 0.05) [32]. Preservation of LBM during significant weight loss could be viewed as a positive outcome in this population as losses of LBM are typical. In a later study that did not use an energy restriction, Demark-Wahnefried et al (2008) [33] assessed the effects of calcium rich diet alone (1200-1500 mg/day), combined with low load resistance training (30 min, 3/wk), or combined with the exercise and a low-fat, high fruit and vegetable intake. No change over time in LBM was seen within or between groups, however when trunk fat was excluded from calculations, the third group experienced less body fat % gain over the 6 month intervention than the other two groups (Change in body fat%: Gp3: +0.2% vs Gp1: +1.7% & Gp2: +1.1%, respectively, p < 0.05). The lack of LBM change is most likely due to the low frequency and low-load callisthenic type resistance training prescribed, which may not have been adequate for optimal stimulation of muscle protein synthesis.

Taken together, LBM loss is most likely prevented by resistance training in women who have been or are being treated for breast cancer. Increases in LBM may be confined to those women who adhere to more intensive exercise protocols [21-23], or in specific sub-populations related to younger age [21] or later stage disease [31]. Dietary energy restriction alone at this stage could be considered contraindicated due to the heightened risk of sarcopenia in this population, while the addition of exercise to an energy restriction may ameliorate this risk [32]. At this stage, no studies have aimed to combine dietary prescription and exercise training to specifically increase LBM. Amino acids and long chain omega-3 fatty acids (LCn-3 FAs) are two potential nutrients that can be targeted to compliment resistance training, yet data is lacking in breast cancer survivor populations.

Advances in nutritional supplementation and support for exercise training in other populations indicate that inclusion of specific nutrients, such as amino acids [39] or possibly long chain omega-3 fatty acids (LCn-3s) [40], may significantly enhance the response of LBM in conjunction with exercise training. To date, studies using dietary interventions in breast cancer have not utilised either of these nutrients to improve LBM outcomes for survivors.

### Omega-3 and body composition change

LCn-3s have been extensively investigated for their ability to preserve LBM in other cancer populations [41,42]. However, the populations typically studied have been those with metastatic or advanced cancer and cachexia. Breast cancer survivors do not experience LBM losses comparable to cachectic populaionts, they are much more like a metabolic syndrome population who undergo slower change often associated with fat gains [43].

Long chain omega-3 fatty acids have been considered as potential body composition modulators with or without dietary energy restriction [44]. However, due to significant heterogeneity in population, body composition measurement, length of trial and dose of LCn-3s some trials have reported no effect [45-50], while others have indicated some effect [51-54]. However, of the studies reporting an improvement of one of more body composition parameters after increased LCn-3s intake, the clinical significance of the changes in LBM seen are minimal [40].

In contrast, recently published data indicate that LCn-3s may have clinical utility as an adjunct to an anabolic stimulus like resistance training [55] or during a hyperaminoacidaemic/hyperinsulinaemic clamp [56,57]. Preliminary evidence suggests that LCn-3s may have a permissive effect on muscle protein synthesis, i.e. reducing anabolic resistance [56,57], and may improve neural activation [55] such that skeletal muscle tissue exhibits a greater response to a given anabolic stimulus. In addition, LCn-3s were seen to improve the ‘anabolic resistance’ found in older populations [56].

The safety of LCn-3 supplementation for doses of up to 4g of EPA & DHA/day has been established as low, with the most common concerns arising in regards to gastrointestinal upset and allergic reactions [58]. There is a theoretical link to an increased risk of bleeding when taken in conjunction with anti-coagulant medication, however this is not considered to be a contraindication in these populations [59].

To the authors’ knowledge, no studies have assessed the effect of exercise training and LCn-3 supplementation alone or together, in women who have had breast cancer. Therefore the current study is aimed at comparing the effects of LCn-3FAs alone, an exercise and nutrition program alone, or a combination of both, and how they influence LBM, QOL and inflammation over 12 and 24 weeks in women who have recently completed treatment for breast cancer. It is hypothesised that the greatest relative LBM gains will occur in the combination group.

## Methods/design

### Primary hypothesis

Breast cancer survivors participating in a specifically designed group based cognitive behaviour therapy nutrition and exercise program and supplementing with 3g LCn-3s will have greater attenuation of LBM after 12 weeks compared to participants taking a supplement of 3g LCn-3s alone.

### Secondary hypotheses

Breast cancer survivors participating in the group based cognitive behaviour therapy nutrition and exercise program taking a supplement of 3g LCn-3s will have improved quality of life after 12 and 24 weeks compared to participants of either the specifically designed nutrition and exercise program or participants taking a supplement of 3g LCn-3s alone.

Breast cancer survivors in the group based cognitive behaviour therapy nutrition and exercise program taking a supplement of 3g LCn-3s acids will have lower levels of inflammation after 12 and 24 weeks compared to participants of either the specifically designed nutrition and exercise program or participants taking a supplement of 3g LCn-3s alone.

### Trial design

In order to determine the relative efficacy of each intervention, the design of the study is a parallel 3-arm randomised controlled trial. The intervention will occur at one site, with recruitment occurring at multiple sites. The primary investigators and the participants allocated to the exercise and nutrition groups (+/- LCn-3s) will be blinded, while the LCn-3 FAs alone group is open label.

### Details of power calculation and sample size

The primary outcome measure is change in lean body mass (LBM) at 12 weeks. Exercise interventions In breast cancer populations have shown LBM increases of 0.7 kg to 1 kg [21-23,27], however other exercise intervention studies have reported attenuation of LBM loss rather than increase [60-62]. Assuming that the minimum difference in LBM across the comparison groups is a mean of 2%, 38 participants per group will be required to detect this difference with 90% power and type 1 error of 5% or less (two-tailed) A total of 114 participants are therefore required. Assuming 10% for attrition and allowing 15% for contingency, 51 subjects per group will need to be recruited to obtain complete data on at least 38 for each group.

The study is sufficiently powered to test the secondary hypotheses. A Bonferroni correction to the Type I error will accommodate the 3 pair-wise comparisons by 2 visits such that p < 0.008 will be considered statistically significant in order to preserve the family-wise Type I error rate of 5% for each secondary outcome.

### Participant recruitment

Women will be recruited through breast cancer oncology centres, radio advertising, social media and breast cancer research registries in Brisbane, Australia. Oncologists, breast care nurses and allied health professionals will inform potential participants of the study during or shortly following treatment (surgery, radiotherapy and/or chemotherapy). Participants will be asked to contact the primary investigator to express official interest in the study and have eligibility determined. Recruitment into the trial will be over 10 to 15 groups of 5 to 15 participants per group. A range of group sizes has been chosen to ensure the maximum number of participants can be recruited as delaying the start of intervention may result in some being excluded due to time elapse since treatment. Within each of these groups, participants will be randomly allocated to one of 3 groups. A record will be kept of the number of participants who have expressed interest to the primary investigator, the number of potential participants who are eligible, ineligible and then finally randomised into the trial. Ethical approval has been received from the participating hospital (UCH HREC: #1034) as well as from the University of Queensland (#2011000079). All participants will provide written informed consent.

### Eligibility criteria

To be included in the study, the women must be >18 years of age; have been diagnosed with early stage breast cancer (Stage 0-IIIa); have successfully completed surgery, radiotherapy and/or chemotherapy more than 6 weeks prior to allow for wound healing and/or shoulder recovery, but not more than12 months post completion of treatment (participants can be currently receiving endocrine and/or herceptin therapy); able to perform moderate intensity physical activity, and have a BMI of >20 and <35 kg/m^2^.

Participants will be excluded if: they have completed their treatment more than 12 months ago; there is presence of metastatic growth or local/distal recurrence of cancer; they have been diagnosed with cardiovascular disease or diabetes; they currently consume, or have in the last 3 months been consuming >1 g of eicosapentanoic acid (EPA) and docosahexanoic acid (DHA) LCn-3s combined per day; or they refuse to be randomly allocated to one of the 3 groups.

Those who are ineligible will be referred to their general medical practitioner with encouragement to pursue appropriate lifestyle recommendations.

### Randomisation

The supplier of the capsules who has no direct contact with the participants will use NQuery Version 7 mixed block design to randomise group order. Participants will be allocated to their group in the order in which they complete baseline assessment. This trial has been registered with Australia New Zealand Clinical Trials Registry: ACTRN12610001005044; and World Health Organisation: Universal trial number is U1111-1116-8520.

### Interventions

The 3 intervention arms include: Daily consumption of LCn-3 FAs (N-3) for 24 weeks; Daily consumption of LCn-3 FAs for 24 weeks plus a supervised 12-week exercise and nutrition group education program (EX+N-3); Daily consumption of placebo oil for 24 weeks and the 12-week program (OO+N-3).

### Long chain omega-3 fatty acid supplementation

Both N-3 and EX+N-3 groups will be prescribed 3 g (1.75 g EPA and 1.25 g DHA) per day taken in five 1 g capsules each containing 0.35 g and 0.25 g for EPA and DHA, respectively. Participants will be recommended to take the dose with a meal, either all at once or spaced throughout the day. Refrigeration of the capsules will be also recommended.

### Placebo supplementation

The OO+N-3 group will be prescribed five 1 g capsules containing olive oil. The placebo capsules are visually identical to the LCn-3 capsules and created by the same vendor.

All capsules were created in the same batch and were sample tested to ensure they contained the indicated dose. All Participants will be asked to avoid ongoing supplementation of any source that contains additional LCn-3s.

### Exercise and nutrition education program

EX+N-3 and OO+N-3 groups will be asked to attend 9 nutrition and exercise sessions over 12 weeks, starting 1 to 10 days after baseline assessment. To ensure adequate group size, both EX+N-3 and OO+N-3 groups will participate in the same sessions. Both participants and primary investigator will be blinded to group allocations, while all capsules will be given out separately to minimise product comparison. The sessions will run for 60-75 minutes at the Wesley Research Institute, Brisbane. The sessions will include 30 to 45 minutes of nutrition education, the remainder of the time is committed to resistance exercise training. The sessions will be facilitated by the Primary Investigator who is an Accredited Practising Dietitian and Accredited Exercise physiologist with relevant clinical experience.

### Semi-supervised exercise program

The supervised exercise sessions are designed as circuit based training sessions. The sessions will be started with active range of motion exercises as a warm up, exercises will then performed and the session completed with specific stretches and flexibility exercises. The exercises include push ups, squats*, lunges, glute bridging, seated row*, shoulder press*, bicep curls* and a series of postural and abdominal exercises (*Exercises marked indicates the use of the Gymstick™). The resistance exercise program is designed to be performed at home using body weight and the Gymstick™, a specialised elastic resistance stick, which has been used in a previous non-cancer population of similarly aged participants [63]. During the supervised sessions feedback will be given regarding technique, exercise progression and modification, and management of injury/discomfort. Participants will be prescribed to reach at least 3 resistance sessions per week including the supervised session, and at least 3 aerobic training sessions each week at home. The participants will be given access to specifically made video material that details the appropriate technique for the majority of the exercises performed in class. The program will be progressed with the addition of new exercises, increased difficulty of exercises by increasing the tension of the Gymstick™, or exercise modification, and through an increase in workload volume (repetitions and sets). Typically, each exercise will be performed as many times as possible in 30-second to one-minute bouts, or until temporary fatigue. This type of workload has been chosen as it is most applicable to home training using body weight and elastic apparatus. In addition, research indicates that reaching temporary fatigue through a low load high-volume protocol results in a similar increase in muscle protein synthesis when compared to a high load protocol with less repetitions [64].

### Nutrition and exercise education program

The nutrition education program was based on a previously validated cognitive behavioural program for weight loss [65] and adapted to focus on healthy food choices for breast cancer survivors. It should be noted that participants will not be given additional advice regarding weight loss or energy restriction throughout the trial. The 9 sessions will include advice on general and breast cancer specific healthy eating, benefits of exercise and practicalities of incorporating healthy habits. Group discussion will be facilitated by the primary investigator to increase practical content. All of the nutrition sessions will be recorded on the Powerpoint slides and provided to the group members via an online portal.

### Side effects of treatment

All participants will be asked to report the appearance of any adverse symptoms that may be related to the exercise program or capsule consumption. If a participant is diagnosed with a recurrence they will be excluded from the data analysis. They will also be advised in how to access ongoing lifestyle treatment in a private setting. If participants report an exacerbation of lymphoedema symptoms they will be referred to a breast cancer specialist physiotherapist for assessment, in addition, they will be advised to cease their upper body resistance training until medical clearance is given to continue as per the ACSM guidelines [66]. For gastro-intestinal upset, or unpredicted reactions that arise during the study period, participants will be asked to cease capsule consumption and advised to seek medical clearance before recommencing.

### Measures

All outcome measures will be performed at baseline, 12 and 24 week time points. The 24 week time point has been included to better understand the practicality of the intervention in terms of maintenance of lifestyle changes after the supervised time. Each assessment period will involve 2 visits to the WRI. Visit 1 measures will include body composition, questionnaires and aerobic fitness testing. Over the next 7 days participants will be asked to: complete the Diet History Questionnaire; wear a uniaxial accelerometer every day; and have a fasting blood sample. At Visit 2, the primary investigator will review the diet history questionnaire, collect the accelerometer and conduct the muscle endurance testing. The progression of participants through the study can be seen in Table 1, with specific timing of outcome measures shown in Table 1.

**Table 1:** Timings for baseline, 12 & 24 week assessments

	**Day 1 – Approx 2 hours**	**Days 2-6**	**Day 7 – approx 15 mins**
**Baseline assessment**	- Consent form and eligibility assessed	- Fasting CRP and EFA test	- Hand in Accel
Week -1 to 0			- Accel
	- LBM,		- Squat
	- Body fat%, Wt, Ht, Waist, Hip		- Push up
	- QOL related questionnaires		- DHQ
			
	- TMill		
	- Handgrip strength		
	- Demographical info		
	- Accel given		
**Mid intervention assessment**	As above except for consent form + Pill counts		
Week 12-13			
**Post-intervention assessment**	Same as mid-intervention assessment		
Week 25-26			

LBM: Lean body mass; Wt: Weight & Ht: Height; Waist & Hip: Girths; QOL: Quality of Life; TMill: Treadmill Sub-max Vo2 test; Accel: 7-day accelerometer; DHQ: Dietary Habits Questionnaire; CRP: Fasting high sensitivity C-reactive protein; LCn-3: Fasting erythrocyte fatty acid analysis; Push up: 60-second push up test; Squat: 60-second squat test.

### Primary outcome measure

#### Body composition

Change over time in percentage lean body mass will be measured using air displacement plethysmograhy (ADP) (BODPOD, COSMED USA Inc). Before each assessment day, the BODPOD scales and air chamber is calibrated as per the manufacturer’s instructions using known weights and volumes, respectively. Air Displacement Plethysmography is considered a valid alternative to hydrodenitometry (or underwater weighing); it is based on the two-compartment model which views the body as two distinct chemical components composed of FM and FFM [67]. ADP was validated against hydrostatic weighting and generated similar result with good precisions when tested repeatedly [67,68]. Amongst health subjects, ADP has been shown to agree well with other laboratory methods including DXA [69,70] and isotope dilution [71]. All measures will be performed by a certified BODPOD assessor.

Participants will be assessed in a non-fasted state. To minimise daily weight variations, participants are measured at a similar time of day (within 60 minutes of initial assessment) at all 3 assessment points. Consumption related weight variations will be controlled by a food and drink record. At the 12 and 24 week assessments, participants will be asked to repeat their intake from the initial assessment. Participants will be provided a lycra suit and hair cap designed for the BODPOD that must be worn during the assessment. Weight is measured with the electronic scale attached to the BODPOD system. Height is measured using a wall mounted stadiometer. The predicted thoracic volume generated by BODPOD software is used for all calculations.

### Secondary outcome measures

#### Quality of life (QOL)

QOL will be measured using the Functional Assessment of Cancer Therapy- Breast + 4 (FACT-B + 4) tool. This tool has been validated for quality of life measurement in cancer survivor populations [72], breast cancer treatment-related arm morbidity [73], measuring QOL change following exercise training [74], and is one of the most widely cited tools in breast cancer research [75] It is comprised of 2 separate tools, the 27-item FACT-G, and the additional 14-item ‘B + 4’ that specifically relates to individuals who have been treated for breast cancer. A five-point Likert scale is utilised (ranging from 0 = ‘not at all’ to 4 = ‘very much’) and includes four subscales (physical, social, emotional, and functional well-being). Higher scores represent better well-being.

#### C-reactive protein

A fasting high sensitivity-CRP will be measured using a latex-enhanced immunoturbidimetric assay of blood serum. Participants will be asked to attend a Healthscope Pathology lab between Day 1 and 7 of each respective assessment period to have a fasted blood sample taken by a qualified lab technician.

#### Body composition

Percentage and total body weight, adipose tissue content will be measured using the BodPod as described above.

### Measure of adherence to capsule intake

Long chain omega-3 fatty acid intake will be accounted for in two ways: erythrocyte LCn-3 FA content, and combination of pill count and diet history questionnaire.

#### Erythrocyte fatty acid analysis

Lipids from red cells are extracted with chloroform methanol mixture. The fatty acids are trans-esterificated to methyl esters with methylation reagent “Meth-Prep 2”. The methylation extract is analysed by gas liquid chromatography method with flame ionisation detection (gas chromatograph Schimadzu G-2010-FID). The proportion of fatty acids content of the erythrocytes expressed as % of total fatty acids.

#### Pill count

All capsule bottles will be handed in at the end of each 12 weeks. All pills not consumed will be counted and recorded over the 24 weeks.

### Measure of adherence to exercise and dietary program

The Active Australia Survey [76], 7-day Uniaxial accelerometry and exercise log during the intervention will be completed for all assessment points in order to determine changes to physical activity. In addition, changes in push-ups and squats will be considered an indirect marker of exercise adherence. Dietary intake will be assessed by an Accredited Practising Dietitian using the Dietary Habits Questionnaire [77]. Additionally, attendance at sessions will be recorded for each group.

A number of other measures will be taken to capture changes in sub-maximal aerobic fitness [78], upper body strength-endurance [78], lower body strength-endurance [78], handgrip strength [79], waist and hip girths [80], fatigue [81], physical function [82,83] and menopausal symptoms [84]. The tools to be used to measure the above are shown in Table 2.

**Table 2:** **Additional measures taken at baseline**, **12**-**weeks & 24 weeks**

**Outcome**	**Tool**
Physical activity & sedentary time	-7-day uniaxial accelerometery
	-Active Australia Questionairre
	-Training log book (Wk 12 & 24)
Changes in aerobic fitness	-Sub-maximal treadmill test (modified Balke)
Muscular endurance	Upper body: 1-min-push up test
	Lower body: 1-min sit-to-stand test
Muscle strength	Grip strength
Dietary intake	Diet history questionnaire
Waist and hip girth	Metal tape measure
Joint pain and physical function	Health Assessment Questionnaire – Disease Index (HAQ-DI)
Menopausal symptoms	Greene Climacteric Scale

### Data analysis

The primary analysis population is intention to treat. The ITT population will include all randomised participants with at least one post-baseline assessment. Analysis will also be performed on the per protocol (PP) population. The PP population will include all participants who were at least 75% compliant to the exercise and nutrition program (as measured by the number of exercise sessions attended) and 70% adherent to pill intake (as measured by pill count returned/diet history questionnaire or by erythrocyte LCn-3 FA).

Baseline demographic and disease related characteristics will be summarised by group as count and percent for categorical variables and number, mean and standard deviation for continuous variables. To compare the three treatment groups at baseline, a chi square test or Fishers exact test will be used for categorical variables and a one way analysis of variance (ANOVA) or Wilcoxon rank-sum test for continuous variables. Baseline demographic and disease-specific characteristics that differ among groups will be considered for covariate adjustments in analysis of all outcomes.

Measurements collected longitudinally will be summarised by group as number, mean and standard deviation, minimum and maximum at each visit (baseline, 12 week, 24 week). Absolute change from baseline will be calculated by subtracting the baseline measurement from the 12 week and 24 week measurements; percent change from baseline will be calculated by dividing the 12 week and 24 week measurements by the baseline measurement. Measurements include: body composition, quality of life, C-Reactive Protein, physical activity and sedentary time, aerobic fitness, muscular endurance, muscle strength, dietary intake, waist and hip girth, joint pain and physical function and menopausal symptoms. All outcome data will be visually inspected for normality. Data with a skewed distribution may be transformed (e.g. log transformation).

The primary outcome of percent change in lean body mass at 12 weeks between the N-3 and EX+N-3 groups will be tested using a contrast in a one-way ANOVA and a p-value <0.05 will be considered statistically significant. Secondary outcomes will be evaluated using mixed models to accommodate the correlation of the repeated measurements taken on an individual over time. For each of the change from baseline outcomes, time (12 week vs 24 week), treatment (N-3, EX+N-3, OO+N-3), the time by treatment interaction and the baseline value of the outcome will be tested as fixed effects with a random subject effect specified. Interactions with p < 0.10 will be retained in the models. Contrasts will be constructed to compare pair-wise differences among the three treatment groups at each time point. Similar mixed models will be fit to evaluate the effect of adjusting for covariates. In addition to the fixed effects for time, treatment, time by treatment and baseline value, covariates at baseline identified as statistically different among the three groups and covariates known or hypothesised to be associated with the particular change from baseline outcome will be evaluated as fixed effects.

## Discussion

This study will further the evidence base in regards to omega-3 and exercise synergies. These findings will be applicable to breast cancer populations and may translate to populations with other chronic diseases. The cessation of LBM loss, fat mass gain and the associated metabolic benefits are an important consideration for women after breast cancer treatment. Thus, the applicability of known and practical lifestyle measures is an important consideration for ongoing management.

## Abbreviations

ADP: Air displacement plethysmography; BMI: Body mass index; CT-scan: Computed tomography; Demo: Demographics; DEXA: Dual-energy X-ray absorptiometry; E-LC: Education program plus LCn-3s; FACT-B + 4: Functional assessment cancer therapy – breast +4 items; Hs-CRP: High sensitivity C-reactive protein; LBM: Lean body mass; LC: 3g/d LCn-3s alone; LCn-3 FAs: Long chain omega-3 fatty acids; MRI: Magnetic resonance imaging; P-LC: 12-week nutrition and exercise education program plus olive oil group; QOL: Quality of life.

## Competing interests

The author’s declare that they have no competing interests.

## Authors’ contributions

CM contributed involved with project design and ethical approval. CM is responsible for overall project management: recruitment, data collection and intervention delivery. JB and SC conceived and sought funding for the trial. JB contributed to study logistics, expertise related to LCn-3 measurement, and intellectually assists with trial management. SC has ongoing intellectual input into research protocol and data analysis. JC provided expertise in regards to statistical analysis and consistency of outcome measure administration and data collection. All authors read and approved the final manuscript.

## Authors’ information

Primary investigator: Cameron McDonald; Accredited Practising Dietitian, Accredited Exercise Physiologist.

## Pre-publication history

The pre-publication history for this paper can be accessed here:

http://www.biomedcentral.com/1471-2407/14/264/prepub
